# Supplementary material for: Molecular architecture of fungal cell walls revealed by solid-state NMR
Source: Nat Commun. 2018 Jul 16;9:2747. doi: 10.1038/s41467-018-05199-0 (PMC6048167; doi:10.1038/s41467-018-05199-0)
Supplement: Supplementary file 1 — Supplementary Information [file 41467_2018_5199_MOESM1_ESM.pdf]

Molecular Architecture of Fungal Cell Walls Revealed by Solid-State NMR

Kang et al.

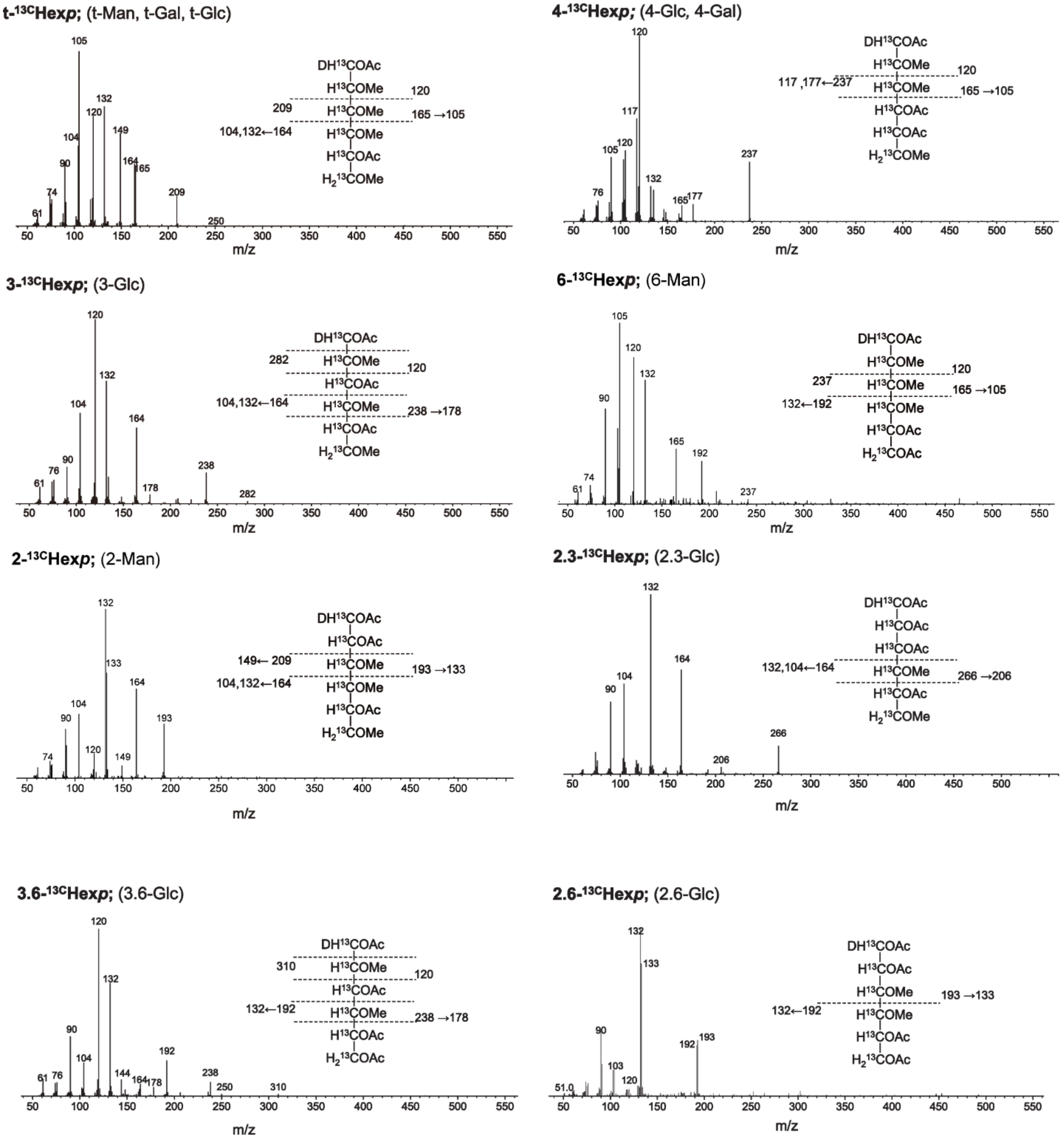

Supplementary Figure 1. EI-MS ion fragmentation of partially methylated alditol acetates of ^{13}C neutral hexoses in fungal cell walls. The proposed glycosyl linkage interpretation of the ^{13}C substituted (~100%) carbohydrates is detailed.

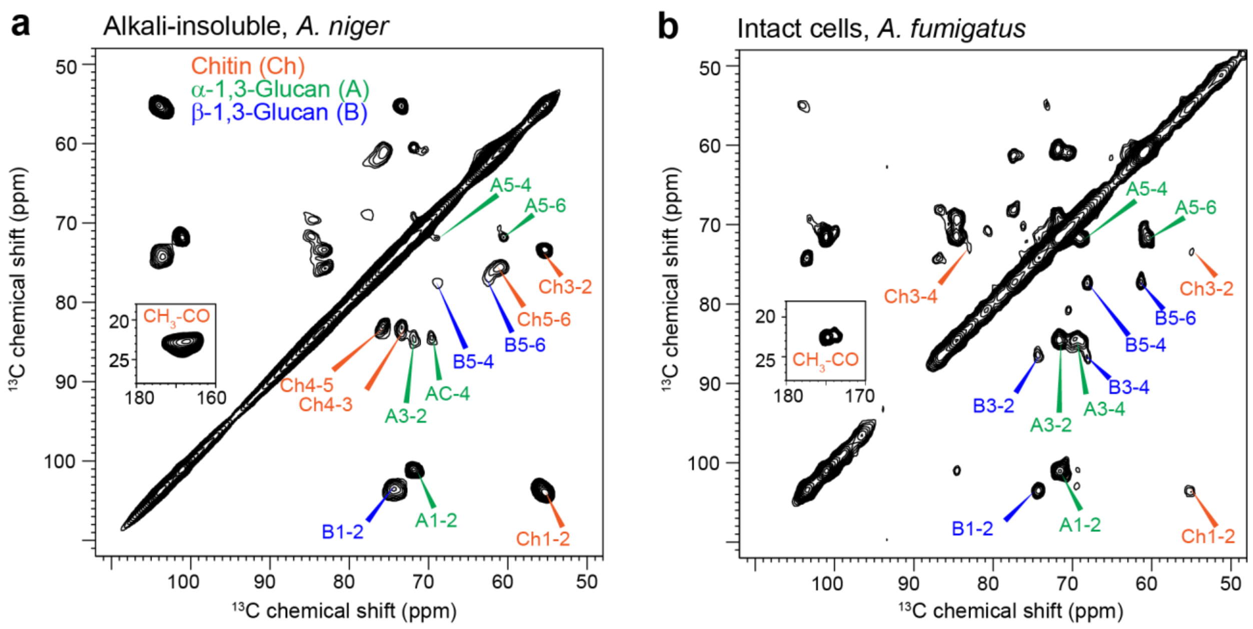

Supplementary Figure 2. Comparison of *A. niger* and *A. fumigatus* cell walls. 2D ^{13}C-^{13}C RFDR spectra of **a**, the alkali-insoluble portion of *A. niger* cell walls, and **b**, intact cells of *A. fumigatus*.

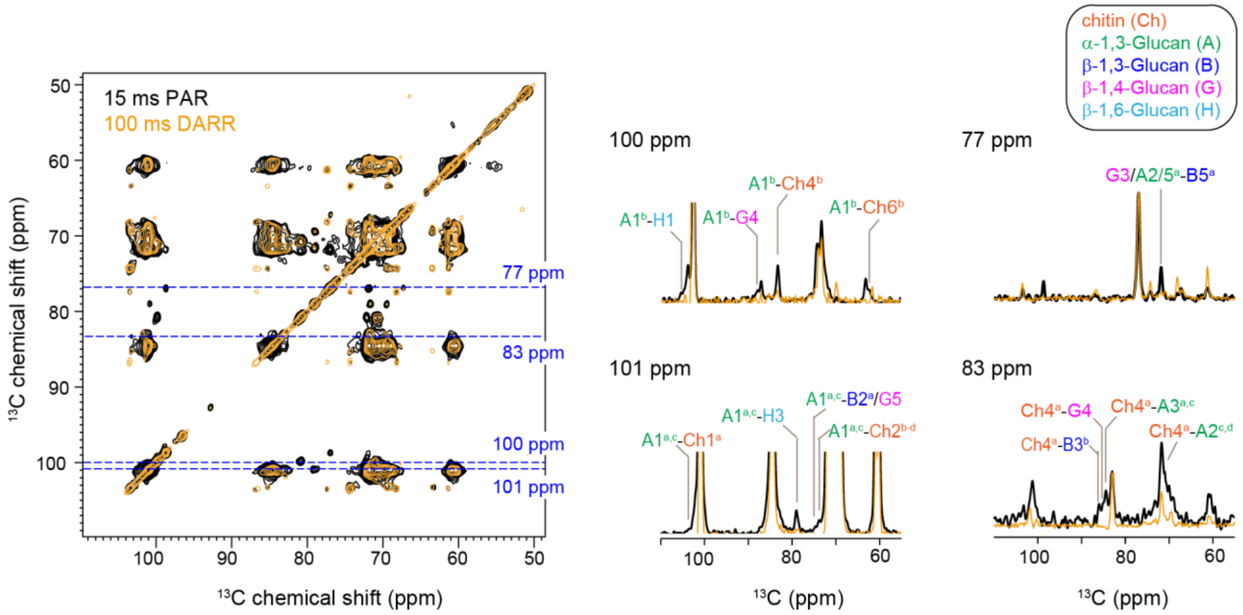

Supplementary Figure 3. ^{13}C cross sections of 2D PAR. The ^{13}C cross sections of 2D PAR (black) and 100 ms DARR (yellow) are overlaid for comparison. Dash lines indicate the positions where ^{13}C cross sections are extracted.

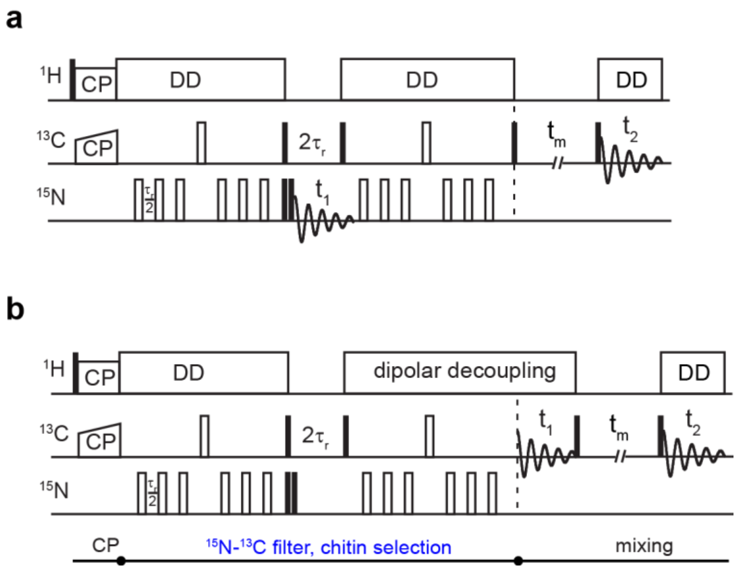

Supplementary Figure 4. DNP Experiments for detecting chitin-glucan interactions. a, ^{15}N-^{13}C correlation experiment. **b**, ^{15}N-^{13}C filtered ^{13}C-^{13}C correlation experiment.

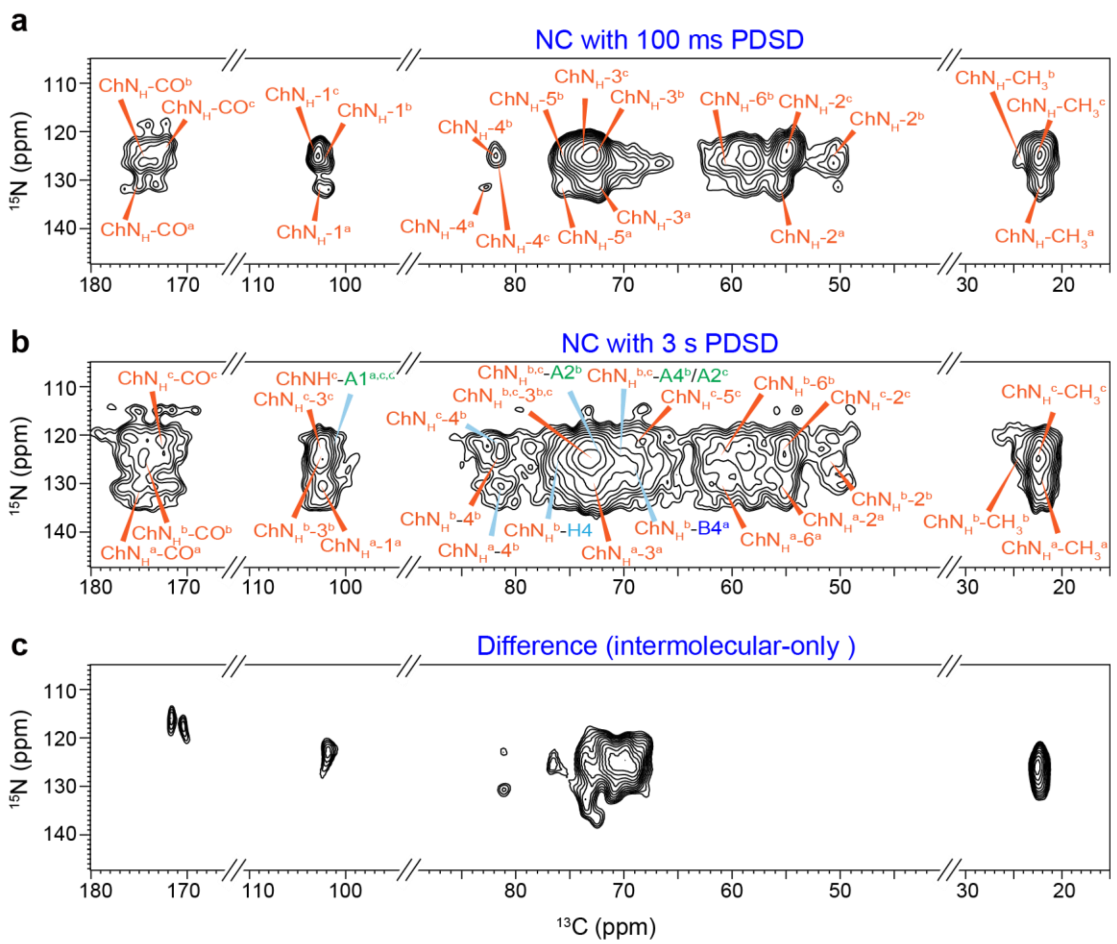

Supplementary Figure 5. Parent spectra for generating intermolecular-only ^{15}N-^{13}C correlation spectrum with improved resolution. a, ^{15}N-^{13}C correlation spectrum with 100 ms mixing times for intramolecular cross peaks. **b,** ^{15}N-^{13}C correlation spectrum with 3 s mixing times for both intermolecular and intramolecular cross peaks. **c,** Spectral subtraction of **b-a** results in a difference spectrum with only long-range intermolecular signals. A Topspin gamma value of -0.65 is used for the subtraction.

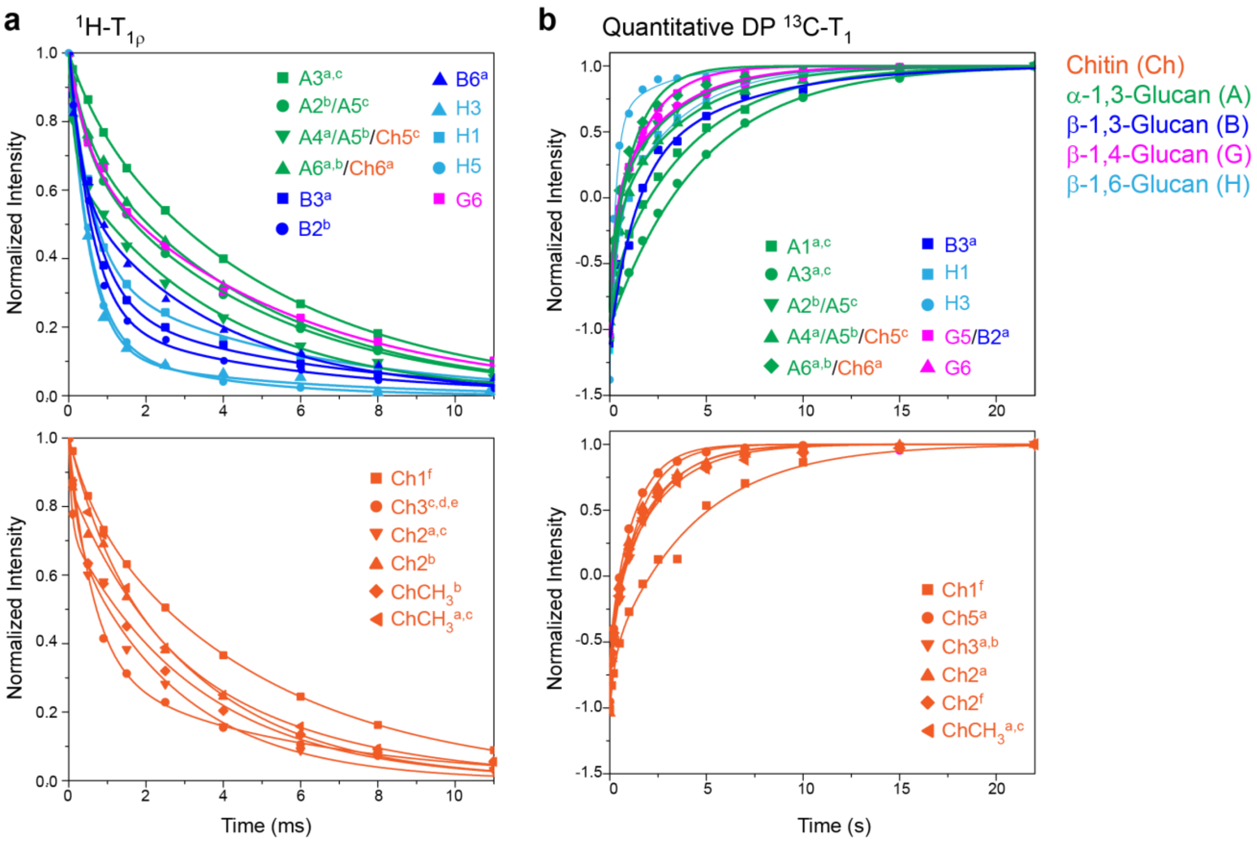

Supplementary Figure 6. NMR relaxation curves of polysaccharides. a, ^{1}H-T$_{1\rho}$ and **b**, ^{13}C-T$_1$ relaxation curves of polysaccharides in intact *A. fumigatus* cell walls. The data are collected on a 400 MHz (9.4 Tesla) spectrometer and best-fits are achieved using single or double exponential equations.

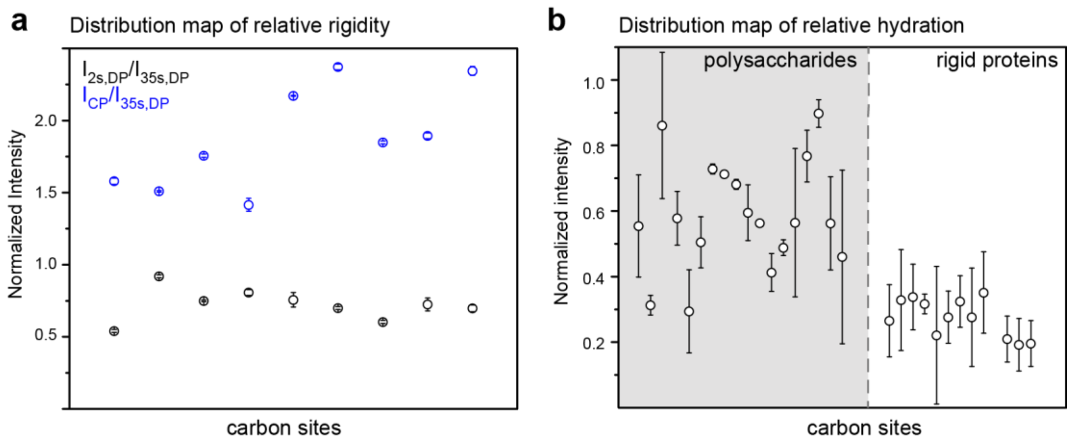

Supplementary Figure 7. NMR analysis of the rigid portion of proteins. a, Comparison of the protein intensity in 1D ^{13}C CP, DP with 2-s recycle delays and DP with 35-s recycle delays. **b**, Comparison of polysaccharide and protein hydration. The normalized intensity is obtained using the intensity ratio between two water-edited spectra with 4-ms and 49-ms ^{1}H mixing time. The 4-ms spectrum contains signals from the well hydrated molecules and the 49-ms spectrum reflects the equilibrium condition. The rigid proteins detected in this CP-based experiment are generally more hydrophobic than the polysaccharides. Error bars are standard deviations propagated from NMR signal-to-noise ratios.

Supplementary Table 1: Glycosyl composition analysis of the neutral sugars in *Aspergillus* cell walls. The intact cell walls of *A. fumigatus* and the alkali-insoluble portion of *A. niger* cell walls are reported.

Glycosyl residue of neutral sugars (mole %)						
Sample	Ara[a]	Man[a]	Gal[a]	Glc[a]		
A. fumigatus	0.3	6.9	14.5	78.3		
A. niger	nd.	2.9	15.2	81.9		
Composition of all sugars (mole %)						
Sample	Ara[b]	Man[b]	Gal[b]	Glc[b]	Chitin[c]	Chitosan[c]
A. fumigatus	0.3	6.2	13.1	70.6	9.0	0.9

[a] Data on neutral sugars are obtained directly from glycosyl composition analysis.

[b] Neutral sugar content rescaled by the NMR data of chitin/glucan and chitin/chitosan ratios.

[c] The amount of nitrogenated sugar in *A. fumigatus* is calculated using the chitin-to-glucan ratio (1 : 7.8) obtained by the area of C1-C2 cross peaks in 2D ^{13}C-^{13}C RFDR spectrum (**Supplementary Fig. 2**) and the chitin-to-chitosan ratio (9 : 1) obtained using 1D ^{15}N spectra (**Fig. 2a**).

Supplementary Table 2. ^{13}C and ^{15}N chemical shifts of polysaccharides in *A. fumigatus* cell walls. Superscripts are used to denote different allomorphs. Underline denotes the ^{13}C connectivity with ambiguity. Weak signals or minor species are indicated using "w." Not applicable (/). Unidentified (-). Unk: unknown.

	C1	C2	C3	C4	C5	C6	CO	CH_3	N	Experimental methods	References
β-1,3-glucan[a]	103.6	74.4	86.4	68.7	77.1	61.3	/	/	/	^{13}C-^{13}C PDSD, ^{13}C CP J-INADEQUATE	Shim et al. 2007<br>Fairweather et al. 2004<br>Hazime Saitô et al. 1979[1-3]
β-1,3-glucan[b] (w)	104.6	75.2	85.9	69.6	78.6	63.0	/	/	/	DNP ^{13}C-^{13}C dipolar-INADEQUATE-PDSD	
β-1,3-glucan[c] (w)	102.6	72.8	84.4	69.7	72.2	60.5	/	/	/	DNP ^{13}C CP J-INADEQUATE	
α-1,3-glucan[a]	101.0	71.9	84.6	69.5	71.7	60.5	/	/	/	^{13}C-^{13}C PDSD, ^{13}C CP J-INADEQUATE	Bhanja et al. 2014 [4]<br>Puanglek et al. 2016 [5]
α-1,3-glucan[b]	99.9	71.0	80.0	70.4	69.7	60.9	/	/	/		
α-1,3-glucan[c]	101.2	70.1	84.5	67.7	71.5	60.5	/	/	/		
α-1,3-glucan[d] (w)	101.5	70.2	86.8	69.3	71.2	62.4	/	/	/	^{13}C CP J-INADEQUATE	
β-1,4-glucan	103.3	69.4	71.7	85.3	74.3	63.4	/	/	/		
β-1,6-glucan	102.6	69.4	79.0	71.6	76.9	67.3					Lowman et al. 2011 [6]
β-1,4-mannan	101.0	69.4	76.2	83.4	77.1	61.6	/	/	/	^{13}C DP J-INADEQUATE	Petkowicz et al. 2001 [7]<br>Marchessault et al. 1990 [8]
arabinan[a]	107.6	81.9	77.1	83.3	69.9	/	/	/	/		Renard et al. 1999[9]
arabinan[b]	108.2	81.7	-	-	-	/	/	/	/		
chitin[a]	103.6	55.5	72.9	83.0	75.7	60.9	174.8	22.6	127.9	^{13}C-^{13}C PDSD, ^{13}C CP J-INADEQUATE, ^{15}N-^{13}C N(CA)CX-DARR	Kono et al. 2004 [10]<br>Heux et al. 2000 [11]<br>Kameda et al. 2004 [12]<br>King et al. 2017 [13]<br>Tanner 1990 [14]
	103.4	56.0	73.9	-	-	-	-	22.2	127.7	RT ^{13}C CP J-INADEQUATE, ^{15}N-^{13}C N(CA)CX-DARR	
	-	55.9	-	-	-	-	-		130.8		
	-	54.7	-	-	-	-	-		131.0		

	-	54.8	-	-	-	-	176.1		129.5		
	102.0	55.2	-	-	-	-	173.0		129.2		
chitin[b]	102.4	51.0	72.9	81.3	74.7	60.3	177.5/174.6/173.0	24.0	123.6	DNP ^{15}N-^{13}C TEDOR, ^{15}N,^{13}C filtered 2D ^{13}C-^{13}C PDSD, ^{15}N-^{13}C N(CA)CX-DARR	
chitin[c]	103.2	55.0	73.4	82.2	69.5	-	172.5	22.6	122.6		
chitin[d] (w)	103.2	56.3	73.9	86.6	-	-	-	-	-	DNP ^{13}C CP J-INADEQUATE	
chitin[e] (w)	103.9	57.1	73.3	87.2	-	-	-	-	-		
chitin[f] (w)	100.6	52.5	71.0	-	-	-	-	-	-		
unk	102.3	71.0	-	-	-	-	-	-	-	^{13}C CP J-INADEQUATE	
unk	100.9	69.5	-	-	-	-	-	-	-		

Supplementary Table 3. The intensity of 65 long-range intermolecular cross peaks. The intensities are relative ratios of the peak area normalized by the integral of a ^{13}C cross section. For non-DNP experiments, a peak higher than 2% is categorized as strong restraints (in bold) and 0.8% for intermediate restraints (underline). For DNP enhanced experiments, given the relatively large values for all cross peaks and the distribution of peak intensities, the thresholds have been increased to 15% and 8% for strong and intermediate restraints, respectively. Error bars are standard deviations propagated from NMR sensitivity.

	atom 1	atom 2	15 ms CC-PAR (%)	3s PDSD (%)	DNP NC-edited CC PDSD (%)	DNP NC with 3s PDSD (%)	DNP 15 ms NN-PAR (%)
β-1,3-glucan-α-1,3-glucan	B5[a]	A3[a,c]	<u>1.05</u>		<u>9.20</u>		
	A1[a,c]	B1[a]/Ch1[a]	**8.11**				
	B5[a]	A4[c]	**7.72**				
	B5[a]	A2[a,b]	**18.54**				
	B5[a]	A6[a,b,c]	**5.57**				
	A6[b]/Ch6[a]	B6[a]	0.35				
	B5[a]	A1[a,c]		**3.91**			
	A1[a,c]	B5[a]		<u>0.88</u>			
	A1[a,c]	B6[a]		<u>0.97</u>			
	A1[a,c]	B5[a]		0.39			
	B1[a]	A2[c]			**15.29**		
Chitin-α-1,3-glucan	A5[a,c]	Ch3[a,c]	**2.22**				
	A6[b]Ch6[a]	Ch6[c]	<u>1.33</u>				
	Ch3[d,e]	A2[c,d]	0.15				
	Ch4[a]	A3[a,c]	**4.63**				
	Ch4[a]	A2[c,d]	**4.62**				
	A1[b]	Ch4[b]	**8.10**				
	A1[b]	Ch6[b]	**3.16**				
	A1[a,c]	Ch4[c]	0.19				
	A1[a,c]	Ch2[b,c,d]	0.36				
	A3[a,c]	Ch2[a,c]		0.48			
	A3[a,c]	Ch1[a,c]		**2.04**			
	A3[a,c]	Ch5[a]		**2.06**			
	Ch2[e]	A3[a,c]			6.35		
	A2[b]	ChCH$_3$[a,c]			4.67		
	ChN$_H$[c]	A1[a,c,d]				<u>10.25</u>	
	ChN$_H$[b,c]	A2[b]				**15.68**	
	ChN$_H$[b,c]	A4[b]/A2[c]				<u>9.87</u>	
Chitin-β-1,3-glucan	Ch4[a]	B3[b]	**3.49**				
	Ch3[d,e]	B5[c]	0.13				
	Ch2[f]	B6[a]			0.49		
	Ch2[d,e]	B6[a]			<u>11.93</u>		
	B2[a]	Ch2[d]			<u>11.54</u>		
	B2[b]	ChCH$_3$[a,c]			3.51		
	B5[a]	ChCH$_3$[a,c]			2.86		
	B5[a]	Ch4[a]			<u>8.92</u>		
	B5[a]	Ch2[d]			7.83		
	Ch4[a]	B6[b]			7.95		
	B1[b]	Ch2[b]			<u>9.53</u>		
	B1[b]	Ch2[d]			<u>13.94</u>		
	ChN$_H$[b]	B4[a]				<u>10.04</u>	
Chitin-Chitin	Ch2[d]	Ch4[a]			6.50		
	Ch4[a]	Ch2[d]			<u>11.47</u>		
	Ch4[a]	Ch2[f]			<u>9.85</u>		

Chitin-Chitin	Ch4[a]	Ch1[f]		2.41		
	Ch4[a]	Ch1[e]		4.71		
	ChN$_H$[a]	ChN$_H$[c,d]				**29.75**
	ChN$_H$[a]	ChN$_H$[e]				**24.39**
	ChN$_H$[c]	ChN$_H$[a]				7.88
	ChN$_H$[a]	Ch4[b]			7.85	
	ChN$_H$[c]	Ch4[b]			8.34	
Chitin-β-1,6-glucan	ChN$_H$[b]	H4			13.33	
	H1	Ch1[f]		1.30		
	H1	Ch1[c,d]		0.18		
	H1	Ch3[c]		3.65		
	H1	Ch2[f]		**15.07**		
	H1	Ch2[a]		12.83		
Chitin-β-1,4-glucan	G6	ChCO[a]		7.58		
	Ch4[a]	G4	**3.57**			
β-1,4-glucan-α-1,3-glucan	A1[b]	G4	1.90			
	A1[a,c]	G5/B2[a]	0.21			
β-1,6-glucan-α-1,3-glucan	A1[a,c]	H1	0.93			
	A1[a,c]	H3	0.98			
α-1,3-glucan - α-1,3-glucan	A1[b]	A1[a,c]	**4.92**			

Supplementary Table 4. The water-edited intensity of polysaccharide cross peaks. The intensity ratios are obtained by comparing the peak intensity in water-edited and control 2D spectra, with further normalization by the most hydrated peak, H2-3. Error bars are standard deviations propagated from NMR signal-to-noise ratios.

Type	Cross peaks	Intensities	Type	Cross peaks	Intensities
Chitin	Ch1-3[a]	0.3 ± 0.1	β-1,4-glucan	G1-4	0.6 ± 0.2
	Ch1-2[a]	0.08 ± 0.07		G1-2	0.60 ± 0.08
	Ch4-2[a]	0.3 ± 0.3		G1-6	0.6 ± 0.2
	Ch5-2[a]	0.1 ± 0.3		G5-4	0.6 ± 0.1
	Ch3-2[a]	0.3 ± 0.3		G5-2	0.7 ± 0.1
	Ch6-2[a]	0.02 ± 0.03		G5-6	0.59 ± 0.07
	Ch2-1[a]	0.02 ± 0.03		G2-1	0.58 ± 0.06
	Ch2-5[a]	0.4 ± 0.4		G2-4	0.61 ± 0.08
	Ch2-4[a]	0.1 ± 0.1		G2-5	0.63 ± 0.08
	Ch2-3[a]	0.20 ± 0.1		G1-1/B1-1[a]	0.57 ± 0.02
	Ch2-6[a]	0.4 ± 0.4		G1-5/B1-2[a]	0.66 ± 0.03
	Ch2-2[a]	0.37 ± 0.05		G5-1/B2-1[a]	0.63 ± 0.03
	Ch5-3[e]	0.04 ± 0.01		G5-5 /B2-2[a]	0.58 ± 0.02
	Ch4-3[f]	0.4 ± 0.1		G2-2/A4-4[a]/H4-4	0.70 ± 0.01
β-1,3-glucan	B1-3[a]	0.7 ± 0.1		G2-6	0.7 ± 0.1
	B1-5[a]	0.6 ± 0.1	α-1,3-glucan	A4-1[a]	0.33 ± 0.02
	B1-4[a]	0.6 ± 0.1		A4-3[a]	0.26 ± 0.04
	B1-6[a]	0.6 ± 0.1		A4-2[a]	0.31 ± 0.02
	B2-3[a]	0.7 ± 0.1		A4-6[b]	0.39 ± 0.06
	B2-5[a]	0.6 ± 0.1		A4-6[a]	0.24 ± 0.03
	B2-4[a]	0.6 ± 0.1		A6-1[a,c]	0.23 ± 0.03
	B2-6[a]	0.6 ± 0.1		A6-3[a,c]	0.20 ± 0.04
	B6-1[a]	0.7 ± 0.2		A6-2[b]/A6-5[c]	0.38 ± 0.01
	B6-3[a]	0.8 ± 0.3		A4-6[a]	0.28 ± 0.02
	B6-5[a]	0.7 ± 0.1		A6-4[b]	0.25 ± 0.01
	B6-2[a]	0.7 ± 0.1		A6-2/5[a]	0.39 ± 0.08
	B6-4[a]	0.7 ± 0.1		A5-2[b]	0.41 ± 0.02
	B6-6[a]/A6-6[a]	0.6 ± 0.0	β-1,6-glucan	H2-3	1.0 ± 0.3
	B4-3[a]	0.8 ± 0.2		H2-6	0.7 ± 0.3

Supplementary Table 5. The relative intensity of polysaccharide signals in 1D ^{13}C CP and DP spectra. Error bars are standard deviations propagated from NMR signal-to-noise ratios.

Assignments	^{13}C (ppm)	$I_{2s,DP}/I_{35s,DP}$	$I_{CP}/I_{35s,DP}$
A1[a,c]	101.1	0.34 ± 0.01	2.06 ± 0.01
A3[a,c]	84.6	0.25 ± 0.01	2.12 ± 0.02
A4[a]	69.9	0.38 ± 0.01	1.80 ± 0.01
A6[a,c]	60.4	0.53 ± 0.01	2.02 ± 0.01
Ch1[b]	102.4	0.79 ± 0.02	1.48 ± 0.03
Ch4[a]	83.1	0.57 ± 0.03	1.74 ± 0.07
Ch4[b]	81.3	0.96 ± 0.05	1.7 ± 0.1
B5[a]	77.3	0.84 ± 0.02	1.42 ± 0.03
B6[a]	61.1	0.86 ± 0.03	1.49 ± 0.01
G6	63.7	0.88 ± 0.02	0.46 ± 0.02
G5	74.8	0.93 ± 0.01	1.45 ± 0.05
H3	78.9	0.94 ± 0.04	1.45 ± 0.05
H6	67.5	0.79 ± 0.01	1.00 ± 0.02
Ch4[c]	82.2	0.85 ± 0.05	1.09 ± 0.06
Ch3[c,d,e]	73.5	0.88 ± 0.01	0.71 ± 0.01
Ch2[e]	57.4	0.85 ± 0.04	0.80 ± 0.04
Ch2[d]	56.9	0.70 ± 0.03	0.95 ± 0.04
Ch2[c]	54.5	0.95 ± 0.02	0.71 ± 0.02
Ch2[f]	52.9	0.76 ± 0.03	0.92 ± 0.04
Ch-CH_3[a,c]	22.8	0.85 ± 0.01	1.16 ± 0.02

Supplementary Table 6. ^{1}H-$T_{1\rho}$ relaxation times of polysaccharides in intact *A. fumigatus* cell walls. Single and double exponential equations are used to fit the data $I(t)=e^{-t/T_{1\rho,b}}$ and $I(t)=ae^{-t/T_{1\rho,a}}+be^{-t/T_{1\rho,b}}$, where b=1-a. Error bars are standard deviations of the fit parameters.

Assignment	^{13}C (ppm)	a	b	$T_{1\rho, a}$(ms)	$T_{1\rho, b}$(ms)
H1	102.8	-	1	-	1.3±0.2
Ch1^{f}	100.6	0.17±0.01	0.83±0.01	0.65±0.04	4.90±0.05
B3^{a}	86.5	-	1	-	1.2±0.1
A3^{a,c}	84.8	0.12±0.02	0.88±0.02	0.7±0.2	5.1±0.1
H3	78.6	-	1	-	0.68±0.04
H5	76.5	-	1	-	0.72±0.04
B2^{b}	75.2	-	1	-	0.92±0.08
Ch3^{c,d,e}	73.7	-	1	-	1.3±0.2
A2^{b}/A5^{c}	71.3	0.29±0.03	0.71±0.02	0.46±0.07	4.7±0.2
A4^{a}/A5^{b}/Ch5^{c}	69.8	0.22±0.03	0.78±0.02	0.42±0.08	4.6±0.2
B6^{a}	61.3	-	1	-	1.6±0.2
A6^{a,b,c}/Ch6^{a,b}	60.7	0.33±0.02	0.67±0.02	0.14±0.02	3.8±0.2
Ch2^{a,c}	54.7	0.14±0.03	0.86±0.02	0.05±0.03	3.2±0.2
Ch2^{b}	51.1	-	1	-	1.7±0.2
Ch-CH_3^{b}	24.0	-	1	-	1.9±0.3
Ch-CH_3^{a,c}	22.3	-	1	-	2.8±0.2

Supplementary Table 7. ^{13}C-T_1 relaxation times of polysaccharides. The data are fit using single and double exponential equations: $I(t)=1-e^{-t/T_{1b}}$ and $I(t)=a(1-e^{-t/T_{1a}})+b(1-e^{-t/T_{1b}})$, where a=1-b. Error bars are standard deviations of the fit parameters.

Assignment	^{13}C (ppm)	a	b	T_{1a} (s)	T_{1b} (s)
H1	102.8	-	1	-	1.54±0.15
A1[a,c]	101.2	-	1	-	2.8±0.9
Ch1[f]	100.6	-	1	-	3.0±0.9
B3[a]	86.5	-	1	-	2.5±0.4
A3[a,c]	84.8	-	1	-	4.3±0.1
H3	78.7	-	1	-	0.4±0.1
Ch5[a]	76.0	0.26±0.05	0.74±0.05	0.17±0.05	1.3±0.1
B2[a]/G5	74.3	-	1	-	1.02±0.07
Ch3[a,b]	72.8	-	1	-	0.95±0.08
A2[b]/A5[c]	71.3	-	1	-	1.6±0.1
A4[a]/A5[b]/Ch5[c]	69.8	-	1	-	1.18±0.09
G6	63.2	-	1	-	1.00±0.09
A6[a,b,c]/Ch6[a]	60.7	0.38±0.07	0.62±0.07	0.17±0.05	1.6±0.2
Ch2[a]	55.4	-	1	-	1.14±0.09
Ch2[f]	52.5	-	1	-	1.0±0.1
Ch-CH_3[a,c]	22.6	-	1	-	1.11±0.09

Supplementary Table 8. The relative intensity of protein signals in 1D ^{13}C CP and DP spectra. Error bars are standard deviations propagated from NMR signal-to-noise ratios.

^{13}C (ppm)	$I_{2s,DP}/I_{35s,DP}$	$I_{CP}/I_{35s,DP}$
33.7	0.54±0.01	1.58±0.02
31.2	0.92±0.01	1.51±0.01
28.3	0.75±0.01	1.76±0.01
21.6	0.81±0.02	1.42±0.05
20.4	0.76±0.05	2.17±0.01
19.3	0.70±0.01	2.37±0.02
17.6	0.60±0.01	1.85±0.01
16.2	0.72±0.05	1.89±0.02
15.6	0.70±0.02	2.34±0.03

Supplementary Table 9. The water-edited intensity of polysaccharide and protein signals. The ratios are obtained by comparing the intensity of each ^{13}C peak in 1D water-edited spectra measured with 4-ms and 49-ms ^{1}H mixing times. Error bars are standard deviations propagated from NMR signal-to-noise ratios.

Type	^{13}C (ppm)	intensity	Type	^{13}C (ppm)	intensity
Polysaccharides	104.0	0.6±0.2	Proteins	32.0	0.3±0.1
	101.7	0.31±0.03		31.4	0.3±0.2
	99.6	0.9±0.2		30.7	0.3±0.1
	87.0	0.58±0.08		30.2	0.32±0.03
	85.2	0.3±0.1		29.3	0.2±0.2
	83.6	0.50±0.08		28.0	0.28±0.08
	81.3	0.73±0.02		26.2	0.32±0.08
	79.8	0.71±0.01		25.9	0.3±0.2
	78.0	0.68±0.02		23.6	0.4±0.1
	74.9	0.59±0.09		20.7	0.21±0.07
	74.2	0.56±0.01		19.5	0.19±0.08
	72.3	0.41±0.06		19.1	0.20±0.07
	70.1	0.49±0.02			
	68.9	0.6±0.2			
	68.0	0.77±0.08			
	67.3	0.90±0.04			
	63.8	0.6±0.1			
	61.6	0.5±0.3			

Supplementary References

1. Shim J. H., *et al.* Antitumor effect of soluble beta-1,3-glucan from Agrobacterium sp. R259 KCTC 1019. *J. Microbiol. Biotechnol.* **17**, 1513-1520 (2007).

2. Fairweather J. K., Him J. L. K., Heux L., Driguez H., Bulone V. Structural characterization by 13C-NMR spectroscopy of products synthesized in vitro by polysaccharide synthases using 13C-enriched glycosyl donors: application to a UDP-glucose:(1→3)-β-d-glucan synthase from blackberry (Rubus fruticosus) *Glycobiology* **14**, 775-781 (2009).

3. Saitô H., Ohki T., Sasaki T. A 13C-nuclear magnetic resonance study of polysaccharide gels. Molecular architecture in the gels consisting of fungal, branched (1 → 3)-β-d-glucans (lentinan and schizophyllan) as manifested by conformational changes induced by sodium hydroxide. *Carbohydr. Res.* **74**, 227-240 (1979).

4. Bhanja S. K., Rut D., Patra P., Sen I. K., Nandan C. K., Islam S. S. Water-insoluble glucans from the edible fungus Ramaria botrytis. *Bioactive Carbohydrates and Dietary Fibre* **3**, 52-58 (2014).

5. Puanglek S., *et al.* In vitro synthesis of linear alpha-1,3-glucan and chemical modification to ester derivatives exhibiting outstanding thermal properties. *Sci. Rep.* **6**, (2016).

6. Lowman D. W., *et al.* New Insights into the Structure of (1 -> 3,1 -> 6)-beta-D-Glucan Side Chains in the Candida glabrata Cell Wall. *Plos One* **6**, (2011).

7. Petkowicz C. L. D., Reicher F., Chanzy H., Taravel F. R., Vuong R. Linear mannan in the endosperm of Schizolobium amazonicum. *Carbohydr. Polym.* **44**, 107-112 (2001).

8. Marchessault R. H., Taylor M. G. 13C CP/MAS NMR spectra of poly-β-D(1 → 4) mannose: mannan. *Can. J. Chem.* **68**, 1992-1995 (1990).

9. Renard C. M. G. C., Jarvis M. C. A cross-polarization, magic-angle-spinning, C-13-nuclear-magnetic-resonance study of polysaccharides in sugar beet cell walls. *Plant Physiol.* **119**, 1315-1322 (1999).

10. Kono H. Two-dimensional magic angle spinning NMR investigation of naturally occurring chitins: Precise H-1 and C-13 resonance assignment of alpha- and beta-chitin. *Biopolymers* **75**, 255-263 (2004).

11. Heux L., Brugnerotto J., Desbrieres J., Versali M. F., Rinaudo M. Solid state NMR for determination of degree of acetylation of chitin and chitosan. *Biomacromolecules* **1**, 746-751 (2000).

12. Kameda T., Miyazawa M., Ono H., Yoshida M. Hydrogen bonding structure and stability of alpha-chitin studied by C-13 solid-state NMR. *Macromolecular Bioscience* **5**, 103-106 (2004).

13. King C., Stein R. S., Shamshina J. L., Rogers R. D. Measuring the Purity of Chitin with a Clean, Quantitative Solid-State NMR Method. *ACS Sustain. Chem. Eng.* **5**, 8011-8016 (2017).

14. Tanner S. F., Chanzy H., Vincendon M., Roux J. C., Gaill F. High-Resolution Solid-State C-13 Nuclear-Magnetic-Resonance Study of Chitin. *Macromolecules* **23**, 3576-3583 (1990).
